# The mammalian LINC complex regulates genome transcriptional responses to substrate rigidity

**DOI:** 10.1038/srep38063

**Published:** 2016-12-01

**Authors:** Samer G. Alam, Qiao Zhang, Nripesh Prasad, Yuan Li, Srikar Chamala, Ram Kuchibhotla, Birendra KC, Varun Aggarwal, Shristi Shrestha, Angela L. Jones, Shawn E. Levy, Kyle J. Roux, Jeffrey A. Nickerson, Tanmay P. Lele

**Affiliations:** 1 Department of Chemical Engineering, University of Florida, Bldg. 723, Gainesville, FL 32611, USA; 2 HudsonAlpha Institute of Biotechnology, Huntsville, AL, 35806, USA; 3 Department of Biology, University of Florida, Cancer and Genetics Research Complex, 2033 Mowry Road, Gainesville, FL 32610, USA; 4 Sanford Children’s Health Research Center, Sanford Research, Sioux Falls, SD 57104, USA, Gainesville, FL 32610, USA; 5 Department of Biological Sciences, University of Alabama in Huntsville, Huntsville, AL 35186, USA; 6 Department of Cell and Developmental Biology, University of Massachusetts Medical School, Worcester, MA 01655, USA

## Abstract

Mechanical integration of the nucleus with the extracellular matrix (ECM) is established by linkage between the cytoskeleton and the nucleus. This integration is hypothesized to mediate sensing of ECM rigidity, but parsing the function of nucleus-cytoskeleton linkage from other mechanisms has remained a central challenge. Here we took advantage of the fact that the LINC (linker of nucleoskeleton and cytoskeleton) complex is a known molecular linker of the nucleus to the cytoskeleton, and asked how it regulates the sensitivity of genome-wide transcription to substratum rigidity. We show that gene mechanosensitivity is preserved after LINC disruption, but reversed in direction. Combined with myosin inhibition studies, we identify genes that depend on nuclear tension for their regulation. We also show that LINC disruption does not attenuate nuclear shape sensitivity to substrate rigidity. Our results show for the first time that the LINC complex facilitates mechano-regulation of expression across the genome.

Mechanical rigidity of the extracellular matrix regulates cell spreading, migration, proliferation and differentiation^1^,^2^,^3^,^4^,^5^. The role of the cytoskeleton and integrin adhesion complexes in mediating effects of substrate rigidity on cell adhesion, spreading and motility has been well studied^6^,^7^,^8^,^9^,^10^,^11^. How this rigidity sensing mechanism ultimately impacts the expression of genes is not well understood. We have recently shown that dynamic cell spreading transmits mechanical stresses to the fibroblast nucleus^12^. Because cells spread less on soft substrates compared to stiff substrates, the resulting differences in nuclear stresses may impact gene expression, but there have been no studies to explore this possibility.

Cytoskeletal stresses are transmitted to the nucleus by the so-called LINC complex which consists of the SUN1/2 (Sad1p, UNC-84) proteins that span the inner nuclear envelope and nesprin proteins embedded in the outer nuclear envelope^13^,^14^,^15^,^16^,^17^. Cytoplasmic domains of nesprins link with the cytoskeleton^13^,^18^,^19^,^20^ while their KASH (Klarsicht, Anc-1, Syne homology) domains bind SUN proteins, which are in turn bound to the lamina. Because the LINC complex can transfer mechanical stresses from the cytoskeleton to the genome^14^,^19^,^20^,^21^,^22^,^23^, here we asked how the sensitivity of genes to substrate rigidity depends on the nucleus-cytoskeleton linkage mediated by the LINC complex. Combined with myosin inhibition studies, we identify genes that depend on nuclear tension for their mechanosensitivity. Our results show for the first time that the LINC complex facilitates mechano-regulation of transcription across the genome.

## Results

### The LINC complex exerts control over the transcriptome

**Figure 1:**
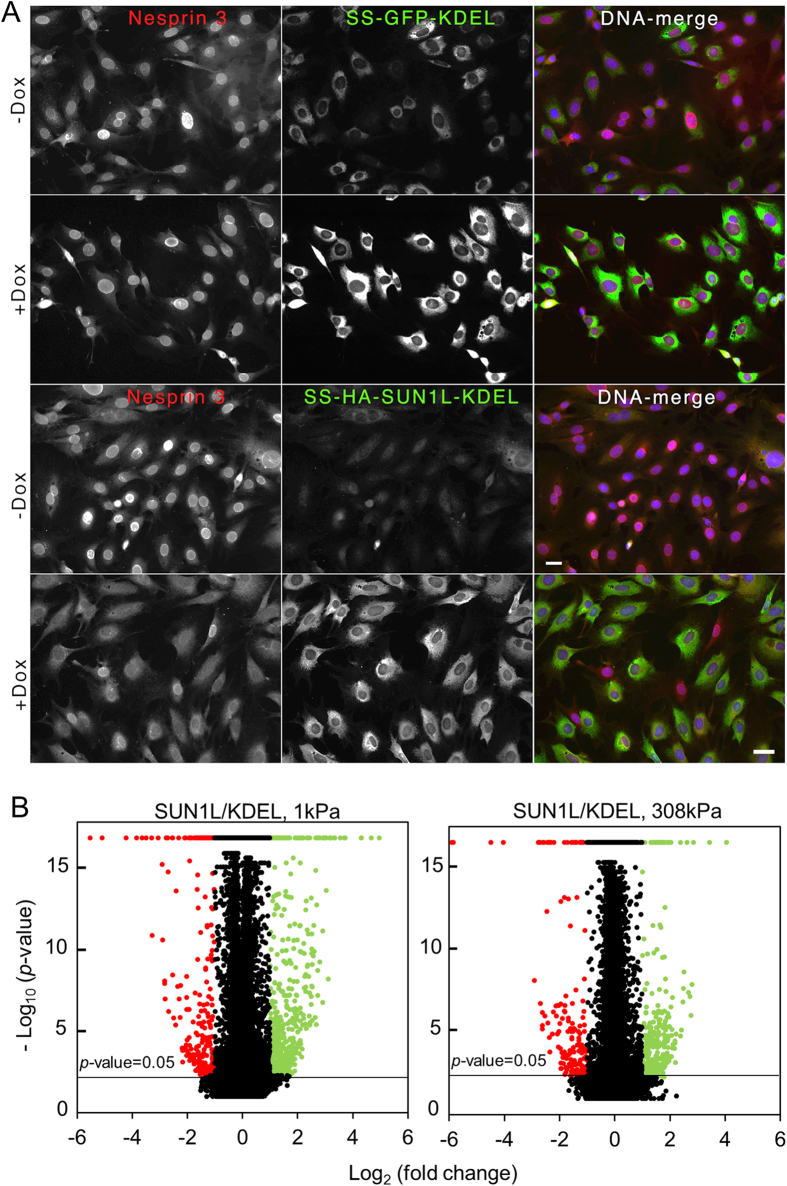
The LINC complex exerts control over the transcriptome. (**A**) Inducible perturbation of the LINC-complex by SUN1L-KDEL. NIH 3T3 TetON cells were induced to express either SS-GFP-KDEL (control, KDEL) or SS-HA-SUN1L-KDEL (SUN1L) by the addition of doxycycline (+Dox). Untreated cells received no doxycycline (−Dox). When expressed, only the SUN1L but not the KDEL control (both green) led to loss of nesprin-3 (red) from the nuclear envelope. DNA is labeled with Hoechst dye (blue) in the merged image. Bar, 30 μm. (**B**) Volcano plots of statistical significance versus fold change between KDEL and SUN1L cells on 1-kPa (left) and 308-kPa (right) substrates; significantly differentially expressed genes are shown in red (downregulated) and green (upregulated). The horizontal line indicates the significance threshold, *p* = 0.05.

We disrupted the LINC complex by inducibly expressing SS-HA-SUN1L-KDEL (signal sequence-HA epitope tag-SUN1 protein lumenal domain-ER retrieval amino acid sequence; hereafter called SUN1L), a dominant-negative construct known to effectively disrupt the LINC complex; GFP-KDEL (hereafter called KDEL) was inducibly expressed to act as controls^17^,^18^,^24^. SUN1L was selected for LINC perturbation instead of GFP-KASH4^24^ due it its increased efficacy in inducible stables (data not shown). We confirmed the successful perturbation of the LINC complex based on observed mislocalization of nesprin-3 at the nuclear envelope compared with KDEL and non-induced SUN1L cells (Fig. 1A). By pulling directly on the nucleus with nanoNewton forces in living, adherent fibroblasts, and quantifying the extent of nuclear deformation, we have previously shown that the elastic linkage between the nucleus and the cytoskeleton is altered in fibroblasts upon expression of SUN1L^25^. We chose NIH 3T3 fibroblasts as they are a well-established model system for cellular sensing of substrate rigidity^9^. To expose cells to a controlled mechanical stimulus, we used the approach in ref. 9 of culturing cells on gels of controlled rigidity (a stiffness of 1 kPa was termed ‘soft’ and that of 308 kPa was termed ‘stiff’). We have recently demonstrated with this system that fibroblasts sense substrate rigidity and not differences in surface ligand presentation^22^. We have also shown that the nucleus is more rounded in its x-z cross-section on soft substrates compared to a more flattened morphology on rigid substrates^22^.

We first asked how transcription is regulated by the LINC complex by statistically comparing mRNA levels quantified by polyadenylated RNA sequencing between KDEL and SUN1L cells on substrates of the same rigidity. RNA sequencing results were validated with qPCR analysis of a small subset of genes (Supplementary Table 20). Differential expression of genes was calculated on the basis of fold changes (using a default cut-off ≥ ±2.0) and the *p*-value of the differentially expressed gene list was estimated with z-score calculations using Benjamini Hochberg^26^ corrections of 0.05 for false-discovery rate. Inducible expression of SUN1L perturbed the levels of a large number of mRNAs on the same substrate (Fig. 1B, Supplementary Tables 1 and 2; see also Spearman correlation plot in Supplementary Figure 1).

### LINC disruption affects genes differently on soft versus stiff substrates

**Figure 2:**
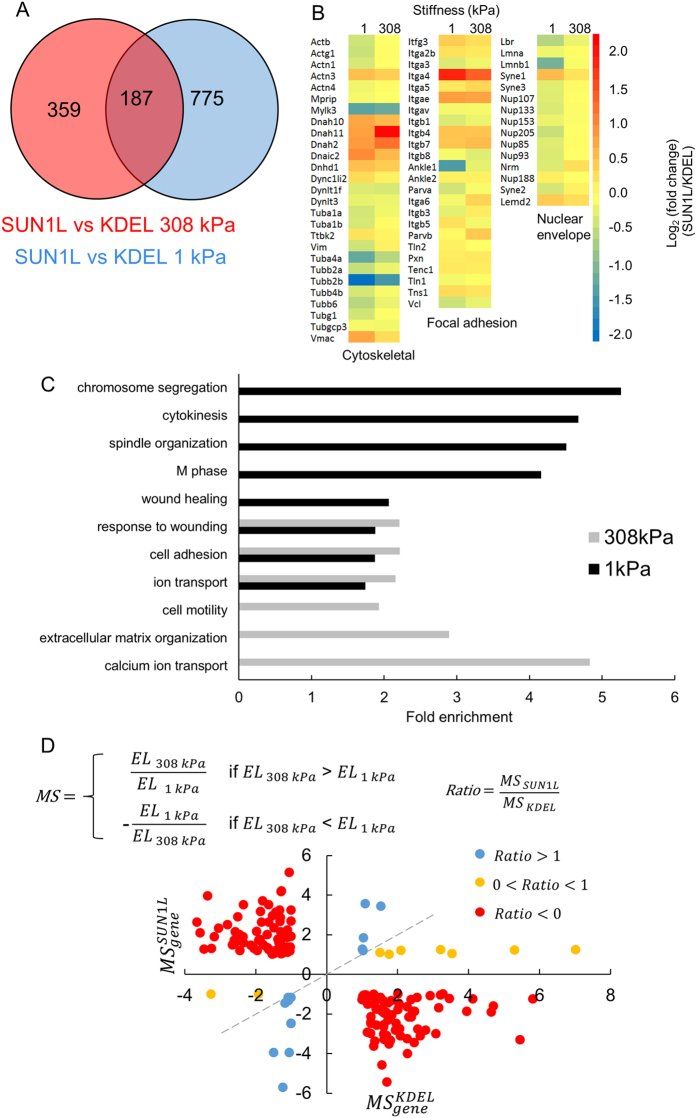
LINC disruption affects genes differently on soft versus stiff substrates. (**A**) Venn diagram summarizing counts of genes that were significantly differentially expressed (*p* < 0.05; |fold change| >2) between KDEL and SUN1L samples on 1 and 308 kPa substrates. (**B**) Heat maps depicting statistically significant gene expression changes upon LINC complex disruption for selected genes for cells cultured on different substrate rigidities. (**C**) Results of a DAVID analysis showing selected significantly enriched biological processes for genes that were differentially expressed upon LINC complex disruption on soft and stiff substrates. (**D**) Upon LINC disruption, a few genes gained mechanosensitivity (ratio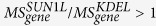, blue solid circles), other genes lost mechanosensitivity (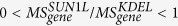, yellow solid circles) and the majority of genes reversed the direction of mechanosensitivity (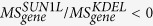, red solid circles). Mechanosensitivity is calculated as the ratio of the expression levels (*EL*) between the two substrate rigidities as shown.

We first confirmed that cells sense substrate rigidity in the assay by comparing the degree of cell spreading on soft and stiff substrates (Table 1); cells spread twice as much on the stiff substrate compared to the soft substrate. Interestingly, on the soft substrate, SUN1L expression significantly altered the mRNA levels of genes coding for focal adhesion proteins (integrins and parvin), cytoskeletal proteins (tubulin and myosin), and nuclear envelope protein lamin B1; but on stiff substrates, different genes were altered (Fig. 2A and B, see also gene lists in Supplementary Tables 3 and 4).

To understand if LINC dependent genes fell in distinct functional classes enriched for specific biological processes, we performed DAVID (*Database for Annotation, Visualization and Integrated Discovery*) functional annotation analysis and Ingenuity Pathway Analysis (IPA). On soft substrates, over-represented biological processes included M phase, chromosome segregation, cell adhesion, ion transport, response to wounding, cytokinesis, spindle organization, and wound healing. On stiff substrates, over-represented processes included calcium ion transport, ion transport, response to wounding, cell adhesion (in agreement with previous phenotypic findings^14^), cell motility (consistent with previous phenotypic findings^17^,^27^,^28^), and extracellular matrix organization (Fig. 2C).

To quantitatively examine the effect of LINC disruption on the mechanosensitivity of individual genes, we applied a two-way Analysis of Variance (ANOVA) to the entire mRNA data set to detect interaction effects between substrate rigidity and SUN1L expression. The analysis revealed 194 genes that exhibited SUN1L-substrate rigidity interactions at a (Bonferroni-corrected) *p* < 0.05 (Supplementary Table 13). Also, Supplementary Table 14 shows genes that depended on substrate rigidity, but were not perturbed by SUN1L expression. We quantified and compared mechanosensitivity (*MS*_*gene*_) of individual genes by defining it as the fold change in mRNA levels of a gene between stiff and soft substrates (for convenience, fold change <1 was recalculated as −1/fold change). We focused only on the genes identified as statistically interacting between substrate rigidity and LINC disruption from the two-way ANOVA analysis. These interacting genes have two attributes: (1) they are mechanosensitive, and (2) their mechanosensitivity (*MS*_*gene*_) is altered upon SUN1L expression. We further classified these genes based on how their mechanosensitivity, *MS*_*gene*_, changed upon LINC disruption, by computing the ratio of mechanosensitivities in SUN1L cells over KDEL cells (
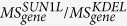
). If 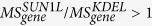, then the gene was classified as having gained mechanosensitivity upon LINC disruption; 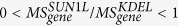 implies a gene that lost mechanosensitivity, while 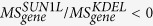 implies a change in the direction of mechanosensitivity.

The major effect of SUN1L expression was to reverse the direction of mechanosensitivity of genes (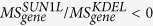) as clear from the large number of interacting genes present in the 2^nd^ and 4^th^ quadrants (Fig. 2D; also see Supplementary Table 13). LINC disruption would generally be expected to eliminate gene mechanosensitivity^29^; that the mechanosensitivity is preserved but its direction is reversed is unexpected.

We analyzed the interacting genes for functional enrichment (Supplementary Table 15). Cell adhesion was the most enriched biological process. This is in agreement with our previously proposed model that LINC complex disruption alters cell adhesion and consequently mechanotransduction^14^.

### Myosin tension-dependent genes that require an intact LINC complex for their regulation

**Figure 3:**
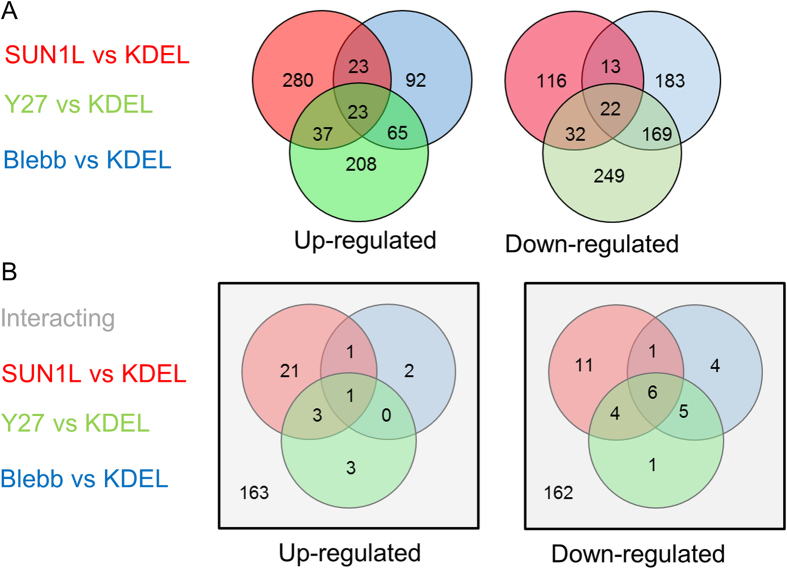
Myosin tension-dependent genes that require an intact LINC complex for their regulation. (**A**) Shown are Venn diagrams summarizing counts of genes that were significantly differentially expressed (*p* < 0.05; fold change >2 (left) and <−2 (right)) in a similar direction upon LINC complex disruption (red), non-muscle myosin II inhibition with blebbistatin (blue), and inhibition of Rho-kinase activity with Y27632 (green). (**B**) Intersection of the list of interacting genes (gray) with each of the three perturbations in A: LINC disruption (red), Y27 (green) and blebbistatin (blue) treatment.

Cellular sensing of substrate rigidity requires myosin activity^22^,^30^,^31^,^32^. As myosin-generated cellular contractile forces can be exerted on the nuclear surface through the LINC complex^14^,^17^,^33^, we asked whether non-muscle myosin II inhibition has similar effects on gene expression as with LINC complex disruption. Because myosin forces are small in cells on the soft substrate^1^,^7^,^34^, we inhibited myosin activity on the stiff substrate with blebbistatin or with Y27632 (a Rho-kinase inhibitor, hereafter called Y27) in control KDEL cells (we have validated myosin inhibition with this approach in NIH 3T3 fibroblasts elsewhere^12^,^27^; see also Supplementary Figure 3). Inhibiting non-muscle myosin II caused significant changes in several genes in KDEL (control) cells (identified by requiring a minimum two-fold change, Supplementary Table 16). We compared this list with the list of genes that change by at least two-fold in response to SUN1L expression in cells cultured on the stiff substrate (Supplementary Table 2). The intersection of these two sets identifies genes that change similarly (in terms of direction and a minimum magnitude) in response to the two perturbations- myosin inhibition and LINC disruption (Fig. 3A and Supplementary Table 17). These myosin tension-dependent genes likely require an intact LINC complex for their regulation.

We performed a further intersection analysis of LINC and myosin-dependent genes with the 194 genes that interact between substrate rigidity and SUN1L expression. We found a few interacting genes that change similarly (in direction and a minimum magnitude) in all three perturbations (LINC disruption, Y27, and blebbistatin treatment), which we further classify as genes that depend on actomyosin forces on the nucleus for their mechanosensitivity (Fig. 3B and Supplementary Table 21).

### Identification of LINC-dependent miRNAs

It is becoming increasingly clear that there are mechanosensitive miRNAs which mediate cellular responses to mechanical stimuli like shear stress^35^ and matrix strain^36^,^37^. We explored the role of the LINC complex in mediating miRNA sensitivity to substrate rigidity by performing miRNA-sequencing. Upon SUN1L expression, the number of miRNAs that changed were four-fold larger on the soft substrate compared with the stiff substrate (compare Supplementary Table 9 and Supplementary Table 10).

We integrated the miRNA and the mRNA datasets from the same samples by performing gene target analysis between experimentally observed differentially expressed mRNAs and miRNAs (Supplementary Tables 11–12 and 18–19) using IPA software. We observed a direct inverse interaction between miRNAs and mRNAs regulating critical pathways such as actin cytoskeleton signaling, Rho family GTPase signaling, paxillin, integrin and FAK signaling and calcium signaling on the soft substrate (Supplementary Figure 2). The LINC complex therefore regulates genes by affecting both mRNA and miRNA levels in fibroblasts.

### Nuclear shape mechano-sensitivity is weakly dependent on the LINC complex

**Figure 4:**
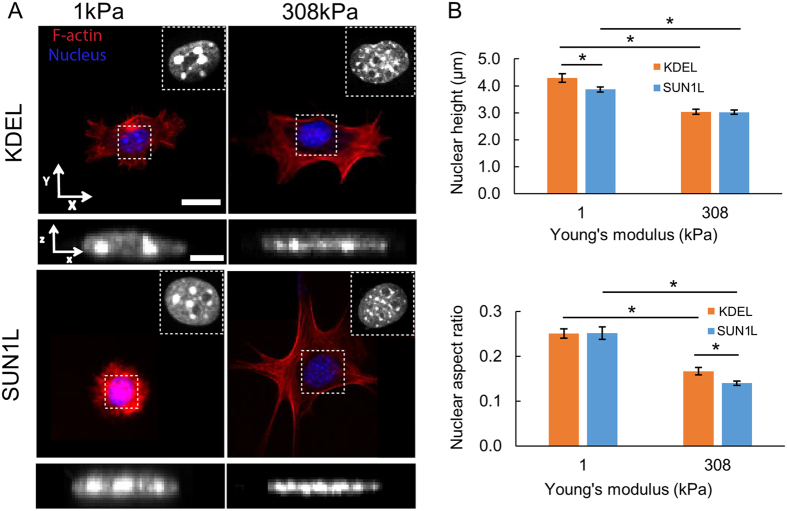
Nuclear shape sensitivity to substrate rigidity is weakly dependent on the LINC complex. (**A**) Confocal images of fixed KDEL (top) and SUN1L (bottom) cells on 1-kPa (left) and 308-kPa (right) substrates, stained with phalloidin for F-actin (red) and Hoechst for DNA (blue), showing the cell and nuclear shapes under various conditions. LINC complex disruption caused minor nuclear shape changes and did not eliminate the nuclear shape sensitivity to substrate rigidity. Bar, 20 μm (*x*-*y* view) and 5 μm (*x*-*z* view). (**B**) Bar graphs of nuclear height (top) and nuclear aspect ratio (bottom) of KDEL and SUN1L cells on 1 and 308 kPa gels showing that disruption of the LINC complex does not alter nuclear shape considerably. Error bars represent standard error of the mean (s.e.m.). *n* ≥ 30 for each condition.

LINC complex disruption alters cytoskeletal force transmission to the nucleus which can affect nucleus shape^25^,^27^,^38^. Thus LINC dependent changes in nucleus morphology might contribute to changes in gene expression through different mechanisms, including changing the location of genes relative to the nucleus periphery^39^,^40^. To test whether changes in overall nuclear shape correlated with LINC-dependent changes in gene expression, we measured nuclear shapes in SUN1L and KDEL cells (Fig. 4A and B) by confocal microscopy according to our previous methods^12^,^22^. We compared *z*-directional height, and major and minor axes in the *x-y* plane (Fig. 4B and Table 1). SUN1L expression caused slight changes in nuclear shape on both substrates (Fig. 4B), but did not eliminate the sensitivity of nucleus shape to substrate rigidity (Fig. 4B and Table 1). Thus, SUN1L expression likely does not affect mechanosensitive gene expression by altering the mechano-sensitivity of nucleus shape.

## Discussion

For many years, it has been hypothesized that the nucleus may be a mechanosensory organelle^41^. Mechanical stresses on the nucleus whether generated within the cytoskeleton or transmitted through the cytoskeleton from the cell membrane, have been shown to induce chromatin remodeling^42^,^43^,^44^, promote DNA repair^45^, promote motion of intranuclear organelles^46^ and cause direct dissociation of protein complexes inside nuclei^47^. These studies suggest that extracellular mechanical cues might regulate gene expression in part by impacting stresses on the nuclear surface. This hypothesis has proven difficult to test, owing to the lack of methods to interfere with nuclear mechanical stresses.

Here we showed that an integrated nucleus-cytoskeleton regulates genome-wide transcriptional response to substrate rigidity. What explains the observed dependence of gene mechanosensitivity on the LINC complex? One possibility is that nuclear shape affects chromatin conformation mechanically (as chromatin is linked to the nuclear lamina^48^,^49^,^50^), and therefore affects accessibility of genes to transcription factors. Healy and coworkers have demonstrated for example that nucleus shapes correlate with the expression of two genes^51^; although a causal linkage between nucleus shape and gene expression has never been directly established. As we showed here, while nucleus shape is sensitive to the rigidity of the substrate, there are only weak effects of SUN1L expression on this sensitivity (Fig. 4A and B). It appears unlikely that the subtle differences in nuclear shape on a given substrate due to SUN1L expression (Fig. 4B and Table 1) causes mechanical modulation of the large number of genes because of chromatin distortion at a global level. These results of course do not rule out the general possibility that nuclear shape changes may impact gene expression in other contexts^51^.

We note that Khatau *et al*. and Kim *et al*. have suggested that the formation of the actin cap governs nuclear shaping, and the actin cap itself is dependent on the LINC complex^52^,^53^. Li *et al*. have shown that nuclear flattening occurs early during cell spreading when apical stress fibers are absent^12^. Moreover, this flattening is independent of myosin activity. They showed that the extent of nuclear flattening is proportional to the extent of cell spreading; any perturbation which prevents cell spreading will prevent nuclear flattening on stiff substrates. In the experiments reported here, SUN1L expression did not affect cell spreading on stiff substrates (Table 1), and predictably nuclear shape was not substantially changed (Table 1 and Fig. 4B). LINC disruption did not necessarily cause rounded nuclear shapes consistent with previous findings^12^.

An alternative possibility is that *direct* mechanical force transfer to the nucleus impacts genome organization and affects gene expression. For example, actin bundles have been shown to indent the nucleus on the apical surface^54^,^55^,^56^, and there is some evidence that actin dependent nuclear deformation may regulate early gene expression^57^. In addition, forces from the cell membrane can be transmitted deep into the nucleus and cause dissociation of protein complexes in the nucleus^47^,^58^. We therefore speculate that local force transfer from the cytoskeleton to the nucleus may enable regulation of at least a sub-set of the genes reported here.

The LINC complex could alternatively regulate transcriptional regulation through non-mechanical mechanisms, such as changes in signaling pathways putatively mediated by the LINC complex. However, our identification of genes that are common to myosin inhibition and LINC disruption suggests a list of genes that likely depend directly on actomyosin tension exerted on the nucleus^14^,^33^,^56^ for their mechanosensitivity. We have previously shown that SUN1L expression softens the integrated nucleus-cytoskeleton in fibroblasts^25^, which likely indicates altered forces on the nucleus. A softer nucleo-cytoskeleton may thus play an important role in gene expression.

Our findings that LINC disruption affects genes in a broad class of cellular processes, including adhesion, motility, ion transport and wound healing highlight the physiological role of the LINC complex. Cytoskeletal, adhesion and nuclear genes are altered upon LINC disruption (Fig. 2B, Supplementary Tables 3 and 4) which is consistent with the primary function that is attributed to the LINC complex- of linking the cytoskeleton to the nucleoskeleton. It is possible that the LINC complex reinforces nucleus-cytoskeletal linkage in response to mechanical cues^59^ which leads to an upregulation of key components of the cytoskeleton and the nucleus. This is also significant because disruptions in the LINC complex can lead to functional mechanical defects at the tissue and organismal levels^60^,^61^.

## Materials and Methods

### Generation of stable cell lines

By PCR cloning we generated pRetroX.Tight.puro retroviral vectors containing either the SUN1L^18^ or KDEL, the latter in which GFP replaced the HA-SUN1L protein to serve as a control. The KDEL sequence was added to target proteins to the rough endoplasmic reticulum, and to keep them from being exported to the Golgi. To generate doxycycline-inducible NIH 3T3 cells, NIH 3T3 Tet-ON 3 G cells (Clontech, Mountain View, CA, USA) were retrovirally transduced prior to selection with 0.5 μg/mL puromycin. SS-GFP-KDEL cells were further enriched by fluorescence sorting on a BD FACSJazz cell sorter. Stable cells were screened by immunofluorescence either in the presence or absence of 1 μg/mL doxycycline induction for 18 hr. Cells were routinely tested for contamination.

### Immunofluorescence Microscopy

The KDEL and SUN1L cells grown on glass coverslips were fixed with 3% (wt/vol) paraformaldehyde/PBS and permeabilized in 0.4% Triton X-100/PBS. Nesprin-3 was detected with mouse anti-nesprin-3 antibody (ab123031, LotGR158650-1, 1:500, Abcam, Cambridge, MA, USA). Rabbit anti-HA (ab9110, LotGR218331-6, 1:1,000, Abcam) was used to detect SUN1L fusion protein. Alexa Fluor-labeled goat anti-rabbit (A11034, Lot1298480, 1:1,000; Invitrogen, Carlsbad, CA, USA) and goat anti-mouse (A11031, Lot11602786, 1:1,000; Invitrogen) were used to visualize the proteins and Hoechst dye 33258 was used to label DNA. Coverslips were mounted using 10% (wt/vol) Mowiol 4–88 (Polysciences, Warrington, PA, USA). Images were obtained using a Nikon Eclipse 90i microscope (40×/0.75 NA Plan Fluor DIC M/N2 objective) and a CCD camera (CoolSNAP HQ2; Photometrics, Tucson, AZ, USA) linked to a workstation running NIS-Element software (Nikon, Melvin, NY, USA).

### Cell culture

The KDEL and SUN1L cells were cultured in Dulbecco’s modified Eagle’s medium with 4.5 g/l glucose (Mediatech, Manassas, VA, USA) supplemented with 10% Donor Bovine Serum (DBS) (Gibco, Grand Island, NY, USA) and 1% penicillin-streptomycin (Mediatech), and were maintained at 37 °C in a humidified environment with 5% CO_2_. Cells were trypsinized, seeded on hydrogel, and induced by adding 1 μg/ml doxycycline (Sigma, St. Louis, MO, USA) for 48 hr to reach a 60–70% confluency prior to lysis or fixation. For myosin inhibition, Y27632 (EMD Millipore, Billerica, MA, USA) and blebbistatin (EMD Millipore) were added to the cell medium at final concentrations of 25 μM and 10 μM, respectively, for 48 hr prior to lysing or fixing.

### Hydrogel preparation and functionalization

Fibronectin-coated (5 μg/ml, BD Biosciences, Franklin, Lakes, NJ, USA) polyacrylamide gels were prepared on glass slides (Fisher, Waltham, MA, USA) as previously described^22^. Two ratios of acrylamide to bis-acrylamide (Fisher), 50:1 and 12.5:1, were used to make gels with Young’s moduli of 1 kPa and 308 kPa, respectively. The Young’s moduli of the polyacrylamide gels were measured using a rheometer as previously described^22^.

### Microscopy and image analysis

Fixed samples were imaged using a Nikon A1 laser scanning confocal microscope (Nikon) with a 60×/1.40 NA oil immersion objective. Images were acquired and processed using NIS Elements software (Nikon). Nuclear height was measured using the full width at half maximum method^62^, and ImageJ software was used to measure the cell area, to fit an ellipse to the nucleus and to calculate its major and minor axis lengths. Nuclear height and aspect ratios were obtained as described previously^12^.

### RNA-seq library preparation, sequencing, and data analysis

After 48 hr of seeding on substrates, cells were lysed using QIAzol Lysis Reagent (Qiagen Sciences, Germantown, MD USA) following the manufacturer’s protocol. Three (3) biological replicates were used for each condition. The cell lysate was immediately stored at −80 °C and transported on dry ice to the Genomic Services Laboratory (HudsonAlpha, Huntsville, AL, USA) for RNA isolation and sequencing. Total RNA containing both mRNA as well as microRNA fractions was extracted from the cell lysate using the miRNeasy Kit with on-column DNase treatment (Qiagen Inc., Valencia, CA, USA) according to the manufacturer’s protocol. The final elution was performed in 30 μl of RNase-free sterile distilled water. The concentration and integrity of the extracted total RNA were estimated using the Qubit 2.0 Fluorometer (Invitrogen) and Agilent 2100 Bioanalyzer (Applied Biosystems, Carlsbad, CA, USA), respectively. RNA samples with RNA integrity numbers (RIN) of at least 7.0 were used for further processing.

Five hundred nanograms of total RNA for samples with RIN values of greater than 7.0 was required for downstream RNA-seq applications. One microliter of a 1:100 dilution of ERCC spike-in consisting of either Mix 1 or Mix 2 (Invitrogen) was added to each sample. Polyadenylated RNAs were isolated using NEBNext Magnetic Oligo d(T)25 Beads. The NEBNext mRNA Library Prep Reagent Set for Illumina (New England BioLabs Inc., Ipswich, MA, USA) was used to prepare individually bar-coded next-generation sequencing expression libraries. Library quality was assessed using the Qubit 2.0 Fluorometer, and the library concentration was estimated by utilizing a DNA 1000 Chip on an Agilent 2100 Bioanalyzer. Accurate quantification for sequencing applications was determined using the qPCR-based KAPA Biosystems Library Quantification Kit (Kapa Biosystems, Inc., Woburn, MA, USA). Each library was diluted to a final concentration of 12.5 nM and pooled in an equimolar ratio prior to clustering. Paired-end sequencing (25 million, 50-bp, paired-end reads) was performed on an Illumina HiSeq2500 sequencer (Illumina, Inc., San Diego, CA, USA).

Post-processing of the sequencing reads from RNA-seq experiments for each sample was performed using the Genomic Services Laboratory unique in-house RNA-seq data analysis pipeline. Briefly, quality control checks on raw sequence data for each sample were performed using FastQC (Babraham Bioinformatics, Cambridge, UK). Raw reads were mapped to the reference mouse genome mm9 using TopHat v2.0^63^. The alignment metrics of the mapped reads were estimated using SAMtools^64^. Aligned reads were imported to the commercial data analysis platform AvadisNGS (Strand Scientifics, CA, USA). After quality inspection, the aligned reads were filtered on the basis of read quality metrics; reads with a base quality score of less than 30, alignment score of less than 95, and mapping quality of less than 40 were removed. Remaining reads were then filtered on the basis of their read statistics; missing mates, translocated, unaligned, and flipped reads were removed. The reads list was then filtered to remove duplicates.

Samples were grouped and transcript abundance was quantified for this final read list using Trimmed Means of M-values as the normalization method^65^. Output data utilized for all subsequent comparisons were summarized as normalized signal values generated by AvadisNGS. Differential expression of genes was calculated on the basis of fold changes (using the default cut-off ≥ ±2.0) observed in comparisons between defined conditions, and the *p*-value of the differentially expressed gene list was estimated by z-score calculations using Benjamini Hochberg^26^ corrections of 0.05 for false-discovery rate. A gene ontology (GO) analysis was performed on the list of differentially expressed mRNAs between samples. Database for Annotation, Visualization and Integrated Discovery (DAVID) v6.7^66^,^67^ was used for this analysis. A functional annotation enrichment analysis was performed; functional annotation clustering based on biological processes, cellular components, and molecular functions demonstrating enrichment in the dataset was examined. Ingenuity Pathway Analysis (IPA, Ingenuity Systems, www.ingenuity.com, Redwood City, CA, USA) software was used to analyze the unique canonical pathways, biological functions, and networks affected by LINC complex disruption on each substrate. Additionally, a two-way analysis of variance (ANOVA) was applied to the entire gene dataset for detecting statistical interaction between substrate rigidity and LINC complex disruption at a Bonferroni-corrected *p* < 0.05. Additional GO-based analyses were performed using DAVID for genes that were significantly affected in the interaction analysis. Sequencing data were deposited in the Gene Expression Omnibus (GEO) repository under series accession numbers GSE77521, GSE77520, GSE 77472.

### MicroRNA (miRNA) Library Preparation, Sequencing and data analysis

Total RNA from each sample was taken into small RNA library preparation protocol using NEBNext Small RNA Library Prep Set for Illumina (New England BioLabs Inc., Ipswich, MA, USA) according to manufacturer’s protocol. Briefly, 3′ adapters were ligated to total input RNA followed by hybridization of multiplex SR RT primers and ligation of multiplex 5′ SR adapters. Reverse transcription (RT) was done using ProtoScript II RT for 1 hour at 50 °C. Immediately after RT reaction, PCR amplification was performed for 15 cycles using LongAmp Taq 2X master mix. Illumina indexed primers were added to uniquely barcode each sample. Post- PCR material was purified using QIAquick PCR purification kit (Qiagen Inc., Valencia, CA, USA). Post-PCR yield and concentration of the prepared libraries were assessed using Qubit 2.0 Fluorometer (Invitrogen, Carlsbad, California, USA) and DNA 1000 chip on Agilent 2100 Bioanalyzer (Applied Biosystems, Carlsbad, CA, USA), respectively. Size selection of small RNA was done using a 3% dye free agarose gel cassettes on Pippin prep instrument (Sage Science Inc., Beverly, MA, USA). Post-size selection yield and concentration of the libraries were assessed using Qubit 2.0 Fluorometer and DNA High sensitivity chip on Agilent 2100 Bioanalyzer, respectively. Accurate quantification for sequencing applications was performed using the qPCR-based KAPA Biosystems Library Quantification kit (Kapa Biosystems, Inc., Woburn, MA, USA). Each library was diluted to a final concentration of 1.25 nM and pooled in equimolar ratios prior to clustering. Single End (SE) sequencing (50 bp) was performed to generate at least 15 million reads per sample on an Illumina HiSeq2500 sequencer (Illumina, Inc., San Diego, CA, USA).

Post processing of the sequencing reads from miRNA-seq experiments from each sample was performed as per the Genomic Services Laboratory unique in-house pipeline. Briefly, quality control checks on raw sequence data from each sample was performed using FastQC (Babraham Bioinformatics, London, UK). Raw reads were imported on a commercial data analysis platform AvadisNGS (Strand Scientifics, CA, USA). Adapter trimming was done to remove ligated adapter from 3′ end of the sequenced reads with only one mismatch allowed, poorly aligned 3′ ends were also trimmed. Sequences shorter than 15 nucleotides length were excluded from further analysis. Trimmed Reads with low qualities (base quality score less than 30, alignment score less than 95, mapping quality less than 40) were removed. Filtered reads were then used to extract and count the small RNA which was annotated with micro RNAs from the miRBase release 20 database. The quantification operation carries out measurement at both the gene level and at the active region level. Active region quantification considers only reads whose 5′ end matches the 5′ end of the mature miRNA annotation. The expression values obtained thus are called ‘raw counts’, candidate miRNAs having less than 10 counts were filtered. Samples were then grouped as identifiers and the differential expression of miRNA was calculated on the basis of their fold change (default cut-off ≥ ±2.0) observed between different groups, *p*-value of differentially expressed miRNA’s was estimated by implementing z-score calculations using Benjamini Hochberg^26^ FDR corrections of 0.05. Gene target analysis for the differentially expressed miRNA was determined on IPA using the miRNA target filter using experimentally validated interactions from TarBase and miRecords, as well as predicted microRNA-mRNA interactions from TargetScan.

### Quantitative RT-PCR (qPCR)

The same RNA used to generate libraries for RNAseq was also used to validate RNAseq results by qPCR. Reverse transcription was performed using the SuperScript IV Reverse Transcriptase (Life Technologies; Grand Island, NY, USA). Quantitative PCR was performed using Taqman primers and probes on a QuantStudio 12 K Flex Real-Time PCR System (Applied Biosystems/Life Technologies). Control qPCR reactions included substitution of No template RT and nuclease free water only, in place of primers and template, to ensure specific amplification in all assays. Dissociation curves for primer sets were evaluated to ensure that no amplicon-dependent amplification occurred. Data generated by qPCR were analyzed using the ΔΔCT method as described by Applied Biosystems (ABI User Bulletin 2, 2001). In this protocol, C_T_ values of both sample and calibrator were normalized to an endogenous reference gene, in this case GAPDH (Supplementary Table 20), for which expression was unaffected by disruption of LINC complex. Genes chosen for qPCR analyses included: POF1B (Premature Ovarian Failure, 1B), YWHAZ (Tyrosine 3-Monooxygenase/Tryptophan 5-Monooxygenase Activation Protein, Zeta) and SNW1 (SNW Domain Containing 1).

## Additional Information

**How to cite this article**: Alam, S. G. *et al*. The mammalian LINC complex regulates genome transcriptional responses to substrate rigidity. *Sci. Rep.* **6**, 38063; doi: 10.1038/srep38063 (2016).

**Publisher's note:** Springer Nature remains neutral with regard to jurisdictional claims in published maps and institutional affiliations.

## Supplementary Material

Supplementary Figures

Supplementary Dataset 1

## Figures and Tables

**Table 1:** Quantification of nuclear shape change between KDEL and SUN1L cells showing only minor shape differences.

Cell condition	KDEL		SUN1L	
Substrate rigidity (kPa)	1	308	1	308
Nuclear height (μm)	4.3 ± 0.2^*^	3.0 ± 0.1	3.9 ± 0.1^*,#^	3.0 ± 0.1
Nuclear x-z aspect ratio	0.25 ± 0.01^*^	0.17 ± 0.01	0.25 ± 0.01^*^	0.14 ± 0.01^#^
Nuclear x-y aspect ratio	0.75 ± 0.01	0.72 ± 0.01	0.81 ± 0.01^*,#^	0.70 ± 0.01
Cell spreading area(μm^2^)	1250 ± 80^*^	2600 ± 200	830 ± 40^*,#^	2300 ± 100

LINC complex disruption did not eliminate the nuclear shape sensitivity to substrate rigidity as revealed by the similar trend in the x-z aspect ratio and cell spreading area between 1 kPa and 308 kPa of KDEL and SUN1L cells. Results reported as mean ± s.e.m. *n* ≥ 30 for each condition.

^*^p < 0.05 comparing to the same cell condition on the 308 kPa substrate using ANOVA.

^#^*p* < 0.05 com*p*aring to KDEL on the same substrate rigidity using ANOVA.

## References

[b1] Discher D. E., Janmey P. & Wang Y. L. Tissue cells feel and respond to the stiffness of their substrate. Science 310, 1139–1143, doi: 10.1126/science.1116995 (2005). 16293750

[b2] Pathak A. & Kumar S. Independent regulation of tumor cell migration by matrix stiffness and confinement. Proc Natl Acad Sci USA 109, 10334–10339, doi: 10.1073/pnas.1118073109 (2012). 22689955 PMC3387066

[b3] Wei S. C. . Matrix stiffness drives epithelial-mesenchymal transition and tumour metastasis through a TWIST1-G3BP2 mechanotransduction pathway. Nat Cell Biol 17, 678–688, doi: 10.1038/ncb3157 (2015). 25893917 PMC4452027

[b4] Paszek M. J. . Tensional homeostasis and the malignant phenotype. Cancer Cell 8, 241–254, doi: 10.1016/j.ccr.2005.08.010 (2005). 16169468

[b5] Peyton S. R. & Putnam A. J. Extracellular matrix rigidity governs smooth muscle cell motility in a biphasic fashion. J Cell Physiol 204, 198–209, doi: 10.1002/jcp.20274 (2005). 15669099

[b6] Gupta M. . Adaptive rheology and ordering of cell cytoskeleton govern matrix rigidity sensing. Nat Commun 6, 7525, doi: 10.1038/ncomms8525 (2015). 26109233 PMC4599139

[b7] Engler A. J., Sen S., Sweeney H. L. & Discher D. E. Matrix elasticity directs stem cell lineage specification. Cell 126, 677–689, doi: 10.1016/j.cell.2006.06.044 (2006). 16923388

[b8] Chan C. E. & Odde D. J. Traction dynamics of filopodia on compliant substrates. Science 322, 1687–1691, doi: 10.1126/science.1163595 (2008). 19074349

[b9] Pelham R. J. & Wang Y. Cell locomotion and focal adhesions are regulated by substrate flexibility. Proc Natl Acad Sci USA 94, 13661–13665 (1997). 9391082 10.1073/pnas.94.25.13661 PMC28362

[b10] Reinhart-King C. A., Dembo M. & Hammer D. A. The dynamics and mechanics of endothelial cell spreading. Biophys J 89, 676–689, doi: 10.1529/biophysj.104.054320 (2005). 15849250 PMC1366566

[b11] Henry S. J., Chen C. S., Crocker J. C. & Hammer D. A. Protrusive and Contractile Forces of Spreading Human Neutrophils. Biophys J 109, 699–709, doi: 10.1016/j.bpj.2015.05.041 (2015). 26287622 PMC4547143

[b12] Li Y. . Moving Cell Boundaries Drive Nuclear Shaping during Cell Spreading. Biophys J 109, 670–686, doi: 10.1016/j.bpj.2015.07.006 (2015). 26287620 PMC4547341

[b13] Burke B. & Roux K. J. Nuclei take a position: managing nuclear location. Dev Cell 17, 587–597, doi: 10.1016/j.devcel.2009.10.018 (2009). 19922864

[b14] Chancellor T. J., Lee J., Thodeti C. K. & Lele T. Actomyosin tension exerted on the nucleus through nesprin-1 connections influences endothelial cell adhesion, migration, and cyclic strain-induced reorientation. Biophys J 99, 115–123, doi: 10.1016/j.bpj.2010.04.011 (2010). 20655839 PMC2895377

[b15] Tapley E. C. & Starr D. A. Connecting the nucleus to the cytoskeleton by SUN-KASH bridges across the nuclear envelope. Curr Opin Cell Biol 25, 57–62, doi: 10.1016/j.ceb.2012.10.014 (2013). 23149102 PMC3578026

[b16] Luxton G. W. & Starr D. A. KASHing up with the nucleus: novel functional roles of KASH proteins at the cytoplasmic surface of the nucleus. Curr Opin Cell Biol 28, 69–75, doi: 10.1016/j.ceb.2014.03.002 (2014). 24704701 PMC4061269

[b17] Lombardi M. L. . The interaction between nesprins and sun proteins at the nuclear envelope is critical for force transmission between the nucleus and cytoskeleton. J Biol Chem 286, 26743–26753, doi: 10.1074/jbc.M111.233700 (2011). 21652697 PMC3143636

[b18] Crisp M. . Coupling of the nucleus and cytoplasm: role of the LINC complex. J Cell Biol 172, 41–53, doi: 10.1083/jcb.200509124 (2006). 16380439 PMC2063530

[b19] Starr D. A. & Fridolfsson H. N. Interactions between nuclei and the cytoskeleton are mediated by SUN-KASH nuclear-envelope bridges. Annu Rev Cell Dev Biol 26, 421–444, doi: 10.1146/annurev-cellbio-100109-104037 (2010). 20507227 PMC4053175

[b20] Simon D. N. & Wilson K. L. The nucleoskeleton as a genome-associated dynamic ‘network of networks’. Nat Rev Mol Cell Biol 12, 695–708, doi: 10.1038/nrm3207 (2011). 21971041

[b21] Lammerding J. . Lamin A/C deficiency causes defective nuclear mechanics and mechanotransduction. J Clin Invest 113, 370–378, doi: 10.1172/JCI19670 (2004). 14755334 PMC324542

[b22] Lovett D. B., Shekhar N., Nickerson J. A., Roux K. J. & Lele T. P. Modulation of Nuclear Shape by Substrate Rigidity. Cell Mol Bioeng 6, 230–238, doi: 10.1007/s12195-013-0270-2 (2013). 23914256 PMC3727663

[b23] Starr D. A. KASH and SUN proteins. Curr Biol 21, R414–415, doi: 10.1016/j.cub.2011.04.022 (2011). 21640895 PMC5518751

[b24] Roux K. J. . Nesprin 4 is an outer nuclear membrane protein that can induce kinesin-mediated cell polarization. Proc Natl Acad Sci USA 106, 2194–2199, doi: 10.1073/pnas.0808602106 (2009). 19164528 PMC2650131

[b25] Neelam S. . Direct force probe reveals the mechanics of nuclear homeostasis in the mammalian cell. Proc Natl Acad Sci USA 112, 5720–5725, doi: 10.1073/pnas.1502111112 (2015). 25901323 PMC4426403

[b26] Benjamini Y. & Hochberg Y. Controlling the false discovery rate - a practical and powerful approach to multiple testing. Journal of the Royal Statistical Society Series B-Methodological 57, 289–300 (1995).

[b27] Alam S. G. . The nucleus is an intracellular propagator of tensile forces in NIH 3T3 fibroblasts. J Cell Sci 128, 1901–1911, doi: 10.1242/jcs.161703 (2015). 25908852 PMC4457156

[b28] Wu J. . Actomyosin pulls to advance the nucleus in a migrating tissue cell. Biophys J 106, 7–15, doi: 10.1016/j.bpj.2013.11.4489 (2014). 24411232 PMC3907209

[b29] Banerjee I. . Targeted ablation of nesprin 1 and nesprin 2 from murine myocardium results in cardiomyopathy, altered nuclear morphology and inhibition of the biomechanical gene response. PLoS Genet 10, e1004114, doi: 10.1371/journal.pgen.1004114 (2014). 24586179 PMC3930490

[b30] Tee S. Y., Fu J., Chen C. S. & Janmey P. A. Cell shape and substrate rigidity both regulate cell stiffness. Biophys J 100, L25–27, doi: 10.1016/j.bpj.2010.12.3744 (2011). 21354386 PMC3043219

[b31] Kraning-Rush C. M., Carey S. P., Califano J. P., Smith B. N. & Reinhart-King C. A. The role of the cytoskeleton in cellular force generation in 2D and 3D environments. Phys Biol 8, 015009, doi: 10.1088/1478-3975/8/1/015009 (2011). 21301071 PMC3138199

[b32] Engler A. J. . Embryonic cardiomyocytes beat best on a matrix with heart-like elasticity: scar-like rigidity inhibits beating. J Cell Sci 121, 3794–3802, doi: 10.1242/jcs.029678 (2008). 18957515 PMC2740334

[b33] Arsenovic P. T. . Nesprin-2G, a Component of the Nuclear LINC Complex, Is Subject to Myosin-Dependent Tension. Biophys J 110, 34–43, doi: 10.1016/j.bpj.2015.11.014 (2016). 26745407 PMC4805861

[b34] Lo C. M., Wang H. B., Dembo M. & Wang Y. L. Cell movement is guided by the rigidity of the substrate. Biophys J 79, 144–152, doi: 10.1016/S0006-3495(00)76279-5 (2000). 10866943 PMC1300921

[b35] Son D. J. . The atypical mechanosensitive microRNA-712 derived from pre-ribosomal RNA induces endothelial inflammation and atherosclerosis. Nat Commun 4, 3000, doi: 10.1038/ncomms4000 (2013). 24346612 PMC3923891

[b36] Rape A. D., Zibinsky M., Murthy N. & Kumar S. A synthetic hydrogel for the high-throughput study of cell-ECM interactions. Nat Commun 6, 8129, doi: 10.1038/ncomms9129 (2015). 26350361 PMC4566157

[b37] Kuo Y. C. . Human mesenchymal stem cells suppress the stretch-induced inflammatory miR-155 and cytokines in bronchial epithelial cells. PLoS One 8, e71342, doi: 10.1371/journal.pone.0071342 (2013). 23967196 PMC3742760

[b38] Dickinson R. B., Neelam S. & Lele T. P. Dynamic, mechanical integration between nucleus and cell- where physics meets biology. Nucleus 6, 360–365, doi: 10.1080/19491034.2015.1090074 (2015). 26338356 PMC4915499

[b39] Meaburn K. J., Gudla P. R., Khan S., Lockett S. J. & Misteli T. Disease-specific gene repositioning in breast cancer. J Cell Biol 187, 801–812, doi: jcb.200909127 [pii]10.1083/jcb.200909127 (2009). 19995938 10.1083/jcb.200909127 PMC2806312

[b40] Meaburn K. J. & Misteli T. Cell biology: chromosome territories. Nature 445, 379–781, doi: 445379a [pii]10.1038/445379a (2007). 17251970 10.1038/445379a

[b41] Wang N., Tytell J. D. & Ingber D. E. Mechanotransduction at a distance: mechanically coupling the extracellular matrix with the nucleus. Nat Rev Mol Cell Biol 10, 75–82, doi: 10.1038/nrm2594 (2009). 19197334

[b42] Iyer K. V., Pulford S., Mogilner A. & Shivashankar G. V. Mechanical activation of cells induces chromatin remodeling preceding MKL nuclear transport. Biophys J 103, 1416–1428, doi: 10.1016/j.bpj.2012.08.041 (2012). 23062334 PMC3471483

[b43] Toh K. C., Ramdas N. M. & Shivashankar G. V. Actin cytoskeleton differentially alters the dynamics of lamin A, HP1α and H2B core histone proteins to remodel chromatin condensation state in living cells. Integr Biol (Camb) 7, 1309–1317, doi: 10.1039/c5ib00027k (2015). 26359759

[b44] Booth E. A., Spagnol S. T., Alcoser T. A. & Dahl K. N. Nuclear stiffening and chromatin softening with progerin expression leads to an attenuated nuclear response to force. Soft Matter 11, 6412–6418, doi: 10.1039/c5sm00521c (2015). 26171741

[b45] Lottersberger F., Karssemeijer R. A., Dimitrova N. & de Lange T. 53BP1 and the LINC Complex Promote Microtubule-Dependent DSB Mobility and DNA Repair. Cell 163, 880–893, doi: 10.1016/j.cell.2015.09.057S0092-8674(15)01327-6 [pii] (2015). 26544937 PMC4636737

[b46] Zhang Q. . Coordinated Dynamics of RNA Splicing Speckles in the Nucleus. J Cell Physiol, doi: 10.1002/jcp.25224 (2015). PMC4755833 26496460

[b47] Poh Y. C. . Dynamic force-induced direct dissociation of protein complexes in a nuclear body in living cells. Nat Commun 3, 866, doi: 10.1038/ncomms1873 (2012). 22643893 PMC3388544

[b48] Osmanagic-Myers S., Dechat T. & Foisner R. Lamins at the crossroads of mechanosignaling. Genes Dev 29, 225–237, doi: 10.1101/gad.255968.114 (2015). 25644599 PMC4318140

[b49] Berk J. M. . The molecular basis of emerin-emerin and emerin-BAF interactions. Journal of cell science 127, 3956–3969, doi: 10.1242/jcs.148247 (2014). 25052089 PMC4163644

[b50] Montes de Oca R., Shoemaker C. J., Gucek M., Cole R. N. & Wilson K. L. Barrier-to-autointegration factor proteome reveals chromatin-regulatory partners. PloS one 4, e7050, doi: 10.1371/journal.pone.0007050 (2009). 19759913 PMC2739719

[b51] Thomas C. H., Collier J. H., Sfeir C. S. & Healy K. E. Engineering gene expression and protein synthesis by modulation of nuclear shape. Proc Natl Acad Sci USA 99, 1972–1977, doi: 10.1073/pnas.032668799 (2002). 11842191 PMC122304

[b52] Khatau S. B. . A perinuclear actin cap regulates nuclear shape. Proc Natl Acad Sci USA 106, 19017–19022, doi: 10.1073/pnas.0908686106 (2009). 19850871 PMC2776434

[b53] Kim D. H. . Actin cap associated focal adhesions and their distinct role in cellular mechanosensing. Sci Rep 2, 555, doi: 10.1038/srep00555 (2012). 22870384 PMC3412326

[b54] Versaevel M., Grevesse T. & Gabriele S. Spatial coordination between cell and nuclear shape within micropatterned endothelial cells. Nat Commun 3, 671, doi: 10.1038/ncomms1668 (2012). 22334074

[b55] Li Q., Kumar A., Makhija E. & Shivashankar G. V. The regulation of dynamic mechanical coupling between actin cytoskeleton and nucleus by matrix geometry. Biomaterials 35, 961–969, doi: 10.1016/j.biomaterials.2013.10.037 (2014). 24183171

[b56] Luxton G. W., Gomes E. R., Folker E. S., Worman H. J. & Gundersen G. G. TAN lines: a novel nuclear envelope structure involved in nuclear positioning. Nucleus 2, 173–181, doi: 10.4161/nucl.2.3.16243 (2011). 21818410 PMC3149877

[b57] Gupta S., Marcel N., Sarin A. & Shivashankar G. V. Role of actin dependent nuclear deformation in regulating early gene expression. PLoS One 7, e53031, doi: 10.1371/journal.pone.0053031 (2012). 23285252 PMC3532443

[b58] Tajik A. . Transcription upregulation via force-induced direct stretching of chromatin. Nat Mater, doi: 10.1038/nmat4729 (2016). PMC5121013 27548707

[b59] Guilluy C. . Isolated nuclei adapt to force and reveal a mechanotransduction pathway in the nucleus. Nat Cell Biol 16, 376–381, doi: 10.1038/ncb2927 (2014). 24609268 PMC4085695

[b60] Meinke P. . Muscular dystrophy-associated SUN1 and SUN2 variants disrupt nuclear-cytoskeletal connections and myonuclear organization. PLoS Genet 10, e1004605, doi: 10.1371/journal.pgen.1004605 (2014). 25210889 PMC4161305

[b61] Burke B. & Stewart C. L. Functional architecture of the cell’s nucleus in development, aging, and disease. Curr Top Dev Biol 109, 1–52, doi: 10.1016/B978-0-12-397920-9.00006-8 (2014). 24947235

[b62] Kuypers L. C., Decraemer W. F., Dirckx J. J. & Timmermans J. P. A procedure to determine the correct thickness of an object with confocal microscopy in case of refractive index mismatch. J Microsc 218, 68–78, doi: 10.1111/j.1365-2818.2005.01457.x (2005). 15817065

[b63] Langmead B., Trapnell C., Pop M. & Salzberg S. L. Ultrafast and memory-efficient alignment of short DNA sequences to the human genome. Genome Biol 10, R25, doi: 10.1186/gb-2009-10-3-r25 (2009). 19261174 PMC2690996

[b64] Li H. . The Sequence Alignment/Map format and SAMtools. Bioinformatics 25, 2078–2079, doi: 10.1093/bioinformatics/btp352 (2009). 19505943 PMC2723002

[b65] Robinson M. D. & Oshlack A. A scaling normalization method for differential expression analysis of RNA-seq data. Genome Biol 11, R25, doi: 10.1186/gb-2010-11-3-r25 (2010). 20196867 PMC2864565

[b66] Huang d. W., Sherman B. T. & Lempicki R. A. Systematic and integrative analysis of large gene lists using DAVID bioinformatics resources. Nat Protoc 4, 44–57, doi: 10.1038/nprot.2008.211 (2009). 19131956

[b67] Huang d. W., Sherman B. T. & Lempicki R. A. Bioinformatics enrichment tools: paths toward the comprehensive functional analysis of large gene lists. Nucleic Acids Res 37, 1–13, doi: 10.1093/nar/gkn923 (2009). 19033363 PMC2615629

