# Supplementary material for: The mammalian LINC complex regulates genome transcriptional responses to substrate rigidity
Source: Sci Rep. 2016 Dec 1;6:38063. doi: 10.1038/srep38063 (PMC5131312; doi:10.1038/srep38063)
Supplement: Supplementary Figures [file srep38063-s1.pdf]

The mammalian LINC complex regulates genome transcriptional responses to substrate rigidity

Samer G. Alam[1], Qiao Zhang[1], Nripesh Prasad[2], Yuan Li[1], Srikar Chamala[3], Ram Kuchibhotla[1], Birendra KC[4], Varun Aggarwal[1], Shristi Shrestha[2, 5], Angela L. Jones[2], Shawn E. Levy[2], Kyle J. Roux[4], Jeffrey A. Nickerson[6], and Tanmay P. Lele[1,*]

[1]Department of Chemical Engineering, University of Florida, Bldg. 723, Gainesville, FL 32611, USA.

[2]HudsonAlpha Institute of Biotechnology, Huntsville, AL, 35806, USA.

[3]Department of Biology, University of Florida, Cancer and Genetics Research Complex,

[4]Sanford Children's Health Research Center, Sanford Research, Sioux Falls, SD 57104, USA.

Gainesville, FL 32610, USA.

[5]Department of Biological Sciences, University of Alabama in Huntsville, Huntsville, AL 35186, USA.

[6]Department of Cell and Developmental Biology, University of Massachusetts Medical School, Worcester, MA 01655, USA.

[*]Address correspondence to: Tanmay P. Lele (tlele@che.ufl.edu).

Department of Chemical Engineering, Bldg 723, University of Florida, Gainesville, FL 32611, USA.

Ph: 352-392-0317

Fax: 352-392-9513

Sequencing data were deposited in the Gene Expression Omnibus (GEO) repository under series accession numbers GSE77521, GSE77520, GSE 77472.

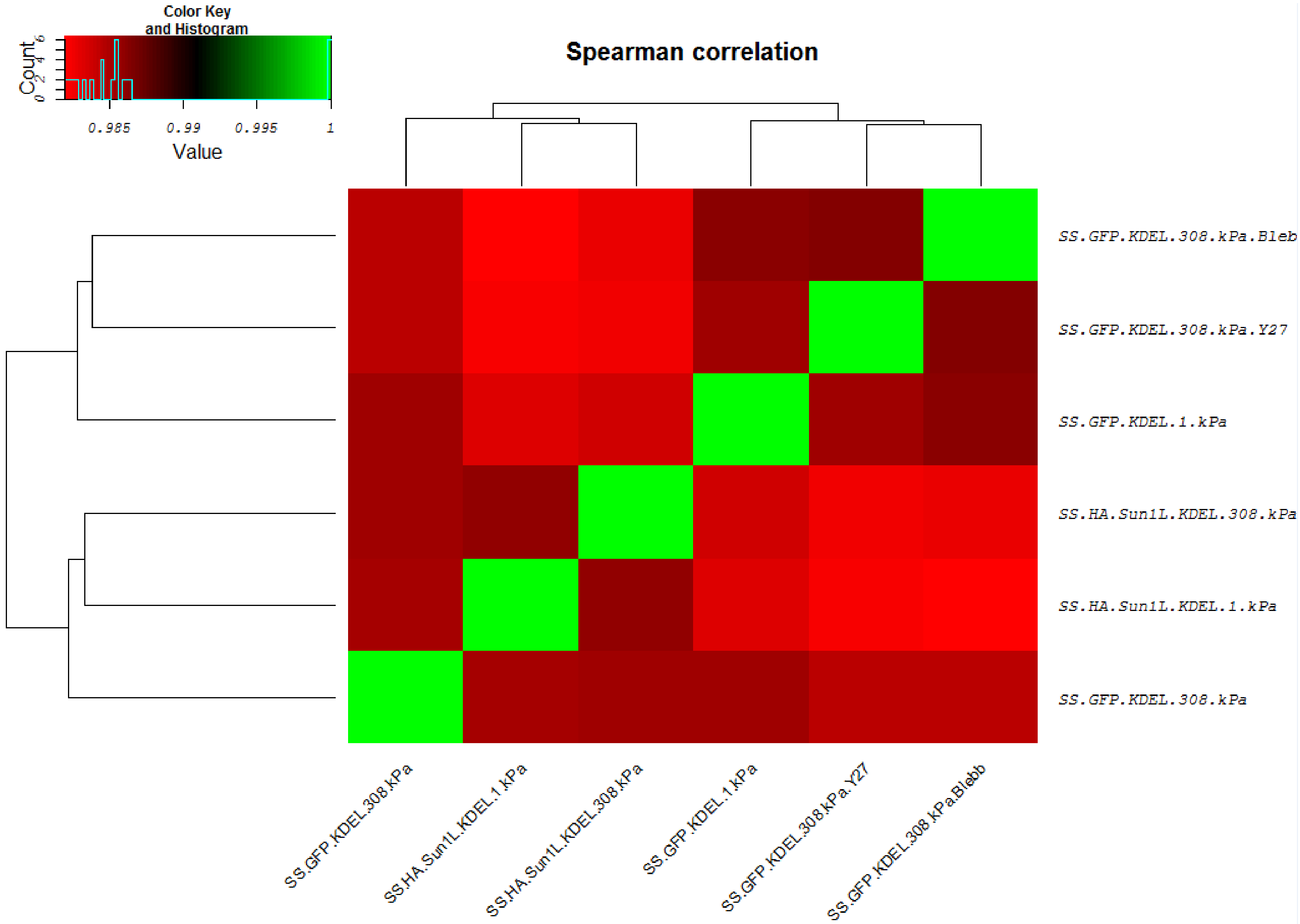

Supplementary Figure 1: Spearman correlation matrix between the indicated samples, measured over gene expression levels of all expressed genes. The matrix is clustered with hierarchical clustering.

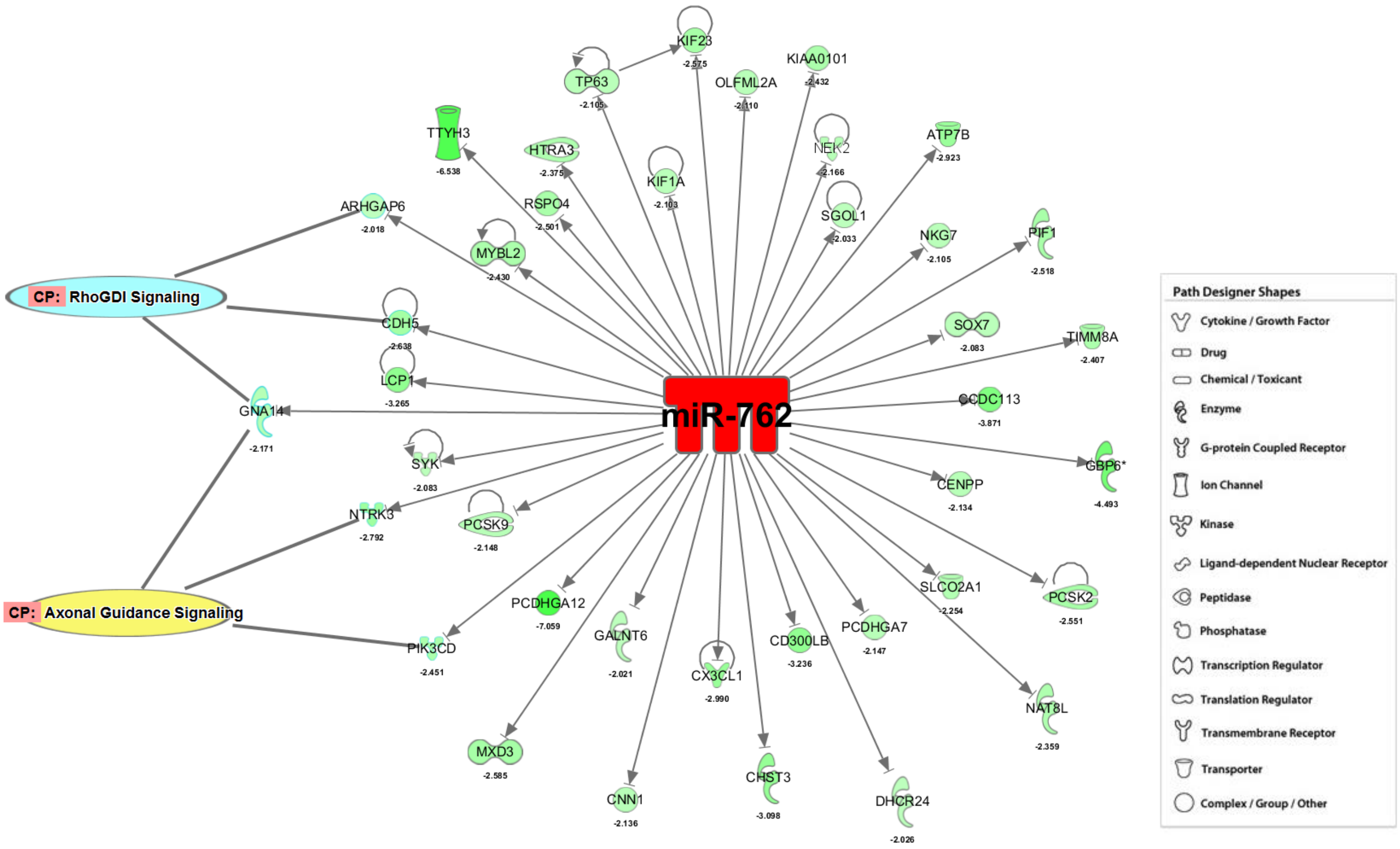

Supplementary Figure 2: An example of Gene Target Analysis interaction network between mRNA and miRNA expression between SUN1L,1kPa and KDEL,1 kPa samples. Green fill indicates down regulated mRNA and red fill indicates Up-regulated miRNA-762. Two Canonical Pathways (CP) that are affected in this network are also shown here.

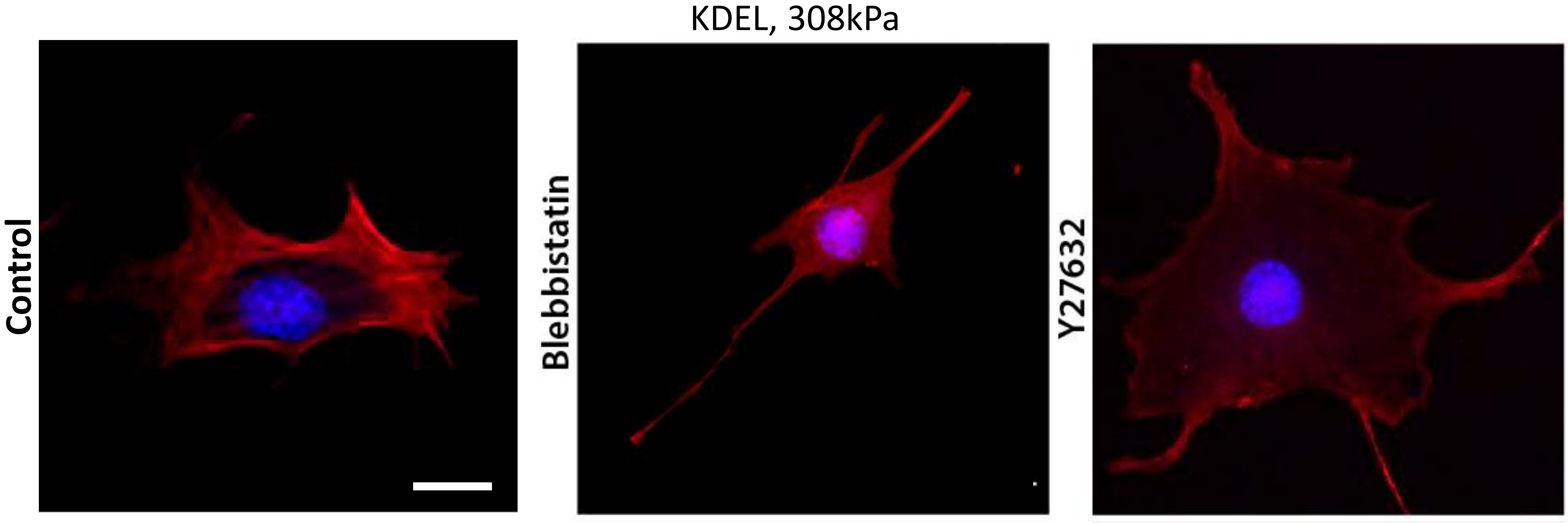

Supplementary Figure 3: Confocal images of untreated, blebbistatin and Y27-treated cells showing clear morphological effects. Red: actin, blue: DNA. Bar, 20µm.
